# Functions of MiRNA-128 on the Regulation of Head and Neck Squamous Cell Carcinoma Growth and Apoptosis

**DOI:** 10.1371/journal.pone.0116321

**Published:** 2015-03-12

**Authors:** Belinda Hauser, Yuan Zhao, Xiaowu Pang, Zhiqiang Ling, Ernest Myers, Paul Wang, Joseph Califano, Xinbin Gu

**Affiliations:** 1 Department of Genetics and Human Genetics, Howard University, Washington, DC, United States of America; 2 Department of Oral Pathology, Howard University, Washington DC, United States of America; 3 Zhejiang Cancer Research Institute, Hangzhou, China; 4 Department of Otolaryngology-Head and Neck Surgery, Howard University, Washington, DC, United States of America; 5 Department of Radiology, Howard University, Washington DC, United States of America; 6 Cancer Center, Howard University, Washington, District of Columbia, United States of America; 7 Departments of Otolaryngology-Head and Neck Surgery, and Head & Neck Research Division, Johns Hopkins University, Baltimore, Maryland, United states of America; Saint Louis University, UNITED STATES

## Abstract

**Background:**

Incidence of head and neck squamous cell carcinoma (HNSCC) has continuously increased in past years while its survival rate has not been significantly improved. There is a critical need to better understand the genetic regulation of HNSCC tumorigenesis and progression. In this study, we comprehensively analyzed the function of miRNA-128 (miR-128) in the regulation of HNSCC growth and its putative targets *in vitro* and *in vivo* systems.

**Methods:**

The function and targets of miR-128 were investigated in human HNSCC cell lines (JHU-13 and JHU-22), which were stably transfected with the miR-128 gene using a lentiviral delivery system. The expression levels of miR-128 and its targeted proteins were analyzed with qRT-PCR, Western blotting and flow cytometry. The binding capacity of miRNA-128 to its putative targets was determined using a luciferase report assay. MTT, colony formation, and a tumor xenograft model further evaluated the effects of miR-128 on HNSCC growth.

**Results:**

We generated two miR-128 stably transfected human HNSCC cell lines (JHU-13^miR-128^ and JHU-22^miR-128^). Enforced expression of miR-128 was detected in both cultured JHU-13^miR-128^ and JHU-22^miR-128^ cell lines, approximately seventeen to twenty folds higher than in vector control cell lines. miRNA-128 was able to bind with the 3′-untranslated regions of BMI-1, BAG-2, BAX, H3f3b, and Paip2 mRNAs, resulting in significant reduction of the targeted protein levels. We found that upregulated miR-128 expression significantly inhibited both JHU-13^miR-128^ and JHU-22^miR-128^ cell viability approximately 20 to 40%, and the JHU-22^miR-128^ tumor xenograft growth compared to the vector control groups.

**Conclusions:**

miR-128 acted as a tumor suppressor inhibiting the HNSCC growth by directly mediating the expression of putative targets. Our results provide a better understanding of miRNA-128 function and its potential targets, which may be valuable for developing novel diagnostic markers and targeted therapy.

## Introduction

Head and neck cancer is one of the cancers with a rising incidence over past 10 years while its survival rate has not been significantly improved [1–3]. More than 90% of head and neck cancers are squamous cell carcinoma (HNSCC), arising in the lining epithelium of the oral cavity, larynx, pharynx, and nasopharynx [4,5]. HNSCC is classified as a complex molecular disease, which develops from dysfunctions of multiple interrelated pathways [1,6]. Moreover, HNSCC has been shown to arise through an accumulation of genetic alterations and there is a need for better understanding of the mechanisms or pathways in responding to the proliferation and apoptosis of HNSCC [7].

MicroRNAs (miRNAs) are key regulators in gene expression that could play a role in HNSCC tumorigenesis. miRNAs are a class of highly conserved small noncoding RNAs (∼22 nucleotides-long), that are known to alter gene expression post-transcriptionally[8]. miRNAs have been shown to act through base pairing with the 3′-untranslated region (3′-UTR) of the target mRNA, resulting in the ability to impede translation of targeted mRNA [9,10]. Blocking of the mRNA leads to the cleavage/or translational repression of the targeted mRNA. Exerting control in the repression of targeted mRNA in combination with other regulatory elements, such as transcription factors have been implicated in dysregulation of critical players in major cellular pathways by mediating cell differentiation, proliferation and survival [11–13]. The dysregulation and dysfunction caused by these unique endogenously expressed miRNAs have been shown to be involved in human diseases and implicated in various types of cancers [8,13]. Increasing evidence has shown that miRNAs have the distinctive ability to function as tumor suppressors or oncogenes [14]. Alterations within the gene transcript have been shown to be critical in tumorigenesis and cancer progression [12,15]. In recent years, comprehensive profiling analysis of miRNAs has been used to identify aberrantly expressed miRNAs [16]. miR-128 is one of the miRNAs, which has been shown to be down-expressed in several types of cancer including prostate cancer, glioma and non-small cell lung cancer, and to inhibit cancer cell growth and invasion when it is constitutively expressed [17–19]. Evidence suggests that miR-128 may play a central role in cellular proliferation by regulating BMI-1, E2fa, and other regulatory element(s) such as transcriptional WEE1-a tyrosine kinase, which phosphorylates CDK1 [19]. In contrast to these studies, Myatt et al. have demonstrated that miR-128 is highly expressed in endometrial cancer. There are still no data available for the expression and function of miR-128 in HNSCC. In the present study, we analyzed the function of miR-128 and its putative targets using HNSCC cells and tumor xenograft models. Our results showed that enforced expression of miR-128 inhibited the HNSCC cell proliferation and tumor xenograft growth by mediating the expression of BMI-1, BAG-2, BAX, H3f3b, and Paip2 mRNAs, suggesting that miR-128 might act as a tumor suppressor.

## Material and Methods

### Chemicals and reagents

Chemicals were of the highest available grade from Sigma (St. Louis, MO). RNeasy Mini Kit was purchased from Qiagen (Gaithersburg, MD) and high-specificity miRNA qRT-PCR detection kit from Stratagene (La Jolla, CA). miR-128 forward primer (5′-TACTGAGCTGTTGGATT-3′) was synthesized at Sigma (Woodlands, TX) according to miRBase: Sequences 12.0.

### Cell lines and culture conditions

Human HNSCC JHU-13 and JHU-22 cell lines were generated by Johns Hopkins University [20,21]. All cells were grown as adhesive monolayers in a humidified atmosphere of 5% CO_2_ in air at 37°C using RPMI 1640 or other suitable medium fortified with 10% fetal bovine serum, and were subculture and seeded at an initial density of 1x10^5^ cells/ml every 3 or 4 days. All experiments were performed with cells in logarithmic phase of growth.

### Quantitation of miRNA expression by quantitative real-time polymerase chain reaction (qRT-PCR)

Total RNA was extracted from the cells using RNeasy Mini Kit (Qiagen) following the manufacturer′s protocol. The total RNA was polyadenylated using *E*. *coli* polyA polymerase, and then 1st-strand cDNA synthesis and quantitative PCR were conducted according to High-Specificity miRNA QRT-PCR Detection kit (Stratagene) protocol. Three controls were performed in the test: (1) a control test without DNA template, and (2) a control test without polyA polymerase to monitoring reagent contamination or false amplification, and (3) an endogenous control test to normalize variations in the amount of cDNA template across samples. The PCR primer sequences of BMI-1, BAX, and miR-128 were designed by Sigma and beta-actin was used as an internal control. The expression level of the miRNA mRNA was determined using the ΔCt method. The average ΔCt of each group was calculated with the formula ΔCt = Ct _target gene_ – Ct _control gene_. ΔΔCt was calculated by ΔΔCt = ΔCt _1_ – ΔCt _2_. The fold-change for miRNA expression level was calculated by 2^−^ΔΔCt. All experiments were performed in triplicate, normalized to either GAPDH or U6, and each value was reported as the level relative to controls.

### Construction of the EGFP-miR-128 expression vector

The EGFP-miR-128 expression vector was constructed with a lentiviral expression system [22]. First, we modified the commercial pLVX-Tight-Puro vector (Clontech, Mountain View, CA) by replacing the P_tight_ promoter with an expression cassette containing a full CMV promoter (P_CMV)_, enhanced green fluorescent protein (EGFP), miRNAs linker, and pre-miR-128-a. The miRNAs linker contained a multiple cloning site. Pre-miR-128-a double strand sequence was designed based on the miRBase:Sequences 12.0 databases. Both pre-miRNA-128-a and pre-miRNA-b formed an identical mature sequence of miR-128. Every step of the vector construction was verified by DNA sequencing. An EGFP control vector was also constructed using the same expression system without the miR-128 gene. Following the manufacturer′s instruction, the lentivirus particles, containing the EGFP-miR-128 vectors or EGFP control vectors, were produced with the lentiphos HT packaging system (Clontech).

### Stable transfection of miRNA-128 in HNSCC cell line

HNSCC cells were pre-seeded in a 6-well plate, cultured overnight, and then infected with 200 μl of the lentivirus for 2 hours followed by the addition of 2 ml of RPMI 1640 medium with 10% fetal bovine serum in each well [22]. After 48 hours, the infected cells were selected with fresh medium containing 5 μg/ml puromycin for 4–5 passages. HNSCC cells stably expressing either EGFP-miR-128 or EGFP alone were generated and the infected cells could be easily viewed under a fluorescence microscope.

### Western immunoblotting assay

Whole-cell lysates were prepared from cell lines with RIPA lysis buffer kit (Santa Cruz Biotechnology, Santa Cruz, CA), and the protein concentrations were quantified using a Bio-Rad protein assay (Bio-Rad, Hercules, CA). Whole-cell proteins (30 μg) were separated on 8% SDS-polyacrylamide gels and transferred to polyvinylidene difluoride membranes (Amersham Corp., Arlington Heights, IL). The membranes were then probed with antibodies against the proteins of H3F3b, BAG-2, Paip2 (Santa Cruz, Santa Cuz, CA), BMI-1 (Millipore, Temecula, CA), EGFR, Bax, and β-actin (Sigma), respectively, for 24 hours. Moreover, membranes were also probed with specific cell cycle, apoptotic and anti-apoptotic antibodies against the following proteins: cyclin A, cyclin B, cyclin D1, p53, caspase 3, caspase 9, NFkb MDM2, BCL2, and BCLxl (Sigma). Washed blots were then incubated with horseradish peroxidase-conjugated anti-mouse antibody (Santa Cruz Biotechnology) for one hour at room temperature. Blots were developed using a peroxidase reaction and visualized with the ECL detection system (Bio-Rad,).

### Flow cytometry assay

The levels of cellular proteins in JHU-22 cells were analyzed by flow cytometry assay. JHU-22^vect^ and JHU-22^miR128^ cells were collected, washed, and fixed in chilled 80% ethanol. The fixed cells were washed twice again with cold phosphate buffered saline (PBS) and then incubated with a solution containing one kind of antibody of Bax, BAG-2, Paip2, EGFR, BMI-1, or H3f3b in the dark for 30 minutes at room temperature. The targeted protein levels (three replicates) were determined with a FACSCalibur flow cytometer and CellQuest Pro software (BD Biosciences, San Jose, CA) by acquiring 10,000 events.

### Construction of 3′-UTR mRNA fragment-luciferase vector (3′-UTR-Lucivector) and luciferase assay

The 3′-UTR-Lucivector was constructed using the phCMV-FSR luciferase reporter vector (Genlantis) with a fragment of mRNA 3′-UTR of BAX (NM_004324 3′-UTR 50–56), BAG-2 (NM_004282 3′-UTR 631–619), PAIP2 (NM_001033112 3′-UTR 42–48), H3f3b (NM_005324 3′-UTR 388–394), or BMI-1 (NM_005180 3′-UTR 481–487), which carry a putative complementary site for mature miR-128. The 293T cells were pre-seeded in a 24-well plate and cultured to 70% confluency for transfection. The binding capacity of miR-128 to its putative targets was evaluated by using a luciferase reporter assay in 293T cells (ATCC, Manassas, VA). Individual 3′-UTR Luci vector and EGFP-miR-128 vector were transfected into 293T cells either alone or together using the calcium phosphate method. Luciferase activity was measured after 24 hours transfection using bioluminescence imaging with a Xenogen IVIS instrument (Caliper Life Sciences, Hopkinton, MA), based on the manufacturer′s protocol.

### Cell viability assay

3-(4, 5-Dimethylthiazol-2-yl)-2, 5-diphenyltetrazolium bromide (MTT) assay was used to estimate the cell viability. JHU-22^vect^ and JHU-22^miR128^ cells were seeded at an initial density of 5000 cells per well in a flat-bottom 96-well cell culture plate and allowed to grow for 48 hours in a humidified 5% CO_2_, 95% air atmosphere in an incubator maintained at 37 ^o^C. Twenty microliters of MTT (5 mg/ml) solution (Sigma) were added to each well followed by 4-hour incubation at 37°C. After the media were removed, 200 μl of dimethyl sulfoxide were added to each well to dissolve the formazan formed. After 30 minutes incubation at room temperature, the plates were scanned with a microplate reader (Bio-Rad) that was set at 560 nm for measuring the absorbance.

### Colony formation assay

This method was used to evaluate the tumorigenic potential of the cells. JHU-22^vect^ and JHU-22^miR128^ cells were seeded at a density of ∼500 cells per well in BD Falcon 6-well tissue culture plates (Palo Alto, CA). The colonies, which formed after nearly nine days, were then stained with 0.1% trypan blue in 50% ethanol. Any colonies containing more than 50 cells were considered to represent a viable clonogenic cell [22]. At least two independent experiments were performed with triplicate test samples.

### Tumor development in athymic nude mice

Four-week-old, male, Balb/c athymic nude mice (Nu/Nu) were obtained from Harlan Sprague Dawley, Inc. (Indianapolis, IN). Mice were housed in temperature-controlled rooms (74 ± 2°F) with a 12-hour alternating light-dark cycle. Approximately 1 x 10^6^ cells were injected subcutaneously into the left (JHU-22^vector^) and right (JHU-22^miR-128^) lower flank of the mice, respectively. The cohorts of 10 mice were housed for 42 days. The mice were then euthanized and the tumor tissues were removed by surgical excision for RNA and protein extraction, and histological studies. The tumor volume and body weight were measured once a week using vernier calipers during the experimental period.

This study was carried out in strict accordance with the recommendations in the Guide for the Care of the Institutional Animal Care and Use Committee at Howard University. The protocol was approved by the Committee on the Ethics of Animal Experiments of the office of regulatory compliance (ORRC) of Howard University (IACUC MED 10–10 R3). All surgery was performed under sodium pentobarbital anesthesia, and all efforts were made to minimize suffering.

### Immunohistochemical staining

Paraffin-embedded tumor xenograft tissues were stained with the Dako Cytomation LSAB2 System-HRP kit following the standard manufacturer′s protocol (DakoCytomation, Carpinteria, CA). Paraffin-embedded tumor specimens were deparaffinized in xylene and rehydrated in graded alcohol. Endogenous peroxidase was blocked using hydrogen peroxide for 20 minutes. The slides were incubated overnight at 4°C with the primary antibodies of proliferating cell nuclear antigen (PCNA), polycomb ring finger oncogene (BMI-1), and cyclin D1 (1:100, EMD, San Diego, CA), respectively, and then incubated with the second antibody for 2 hours at room temperature. Reaction with streptavidin-HRP was carried out for 20 minutes at room temperature. The development time with DAB solution depended on the sample conditions. The stained slides were visualized on a microscope. Images were captured with BioRad ChemIDoc XRS system with imaging software. For quantitative analysis, ten representative high magnification fields were selected for each slide. The expression levels of PCNA, BMI-1 and cyclin D1 were presented as percentage of positive cells in the total number of cells. The expression levels of other proteins were not examined because of limited samples of some small tumor xenografts.

### Statistical analysis

Quantitative data were presented as the mean ± S.D. T-tests were used to determine statistical significance. Differences were considered significant at *P* < 0.05.

## Results

### Establishment of miRNA-128 stably expressed HNSCC cell lines

**Fig 1:**
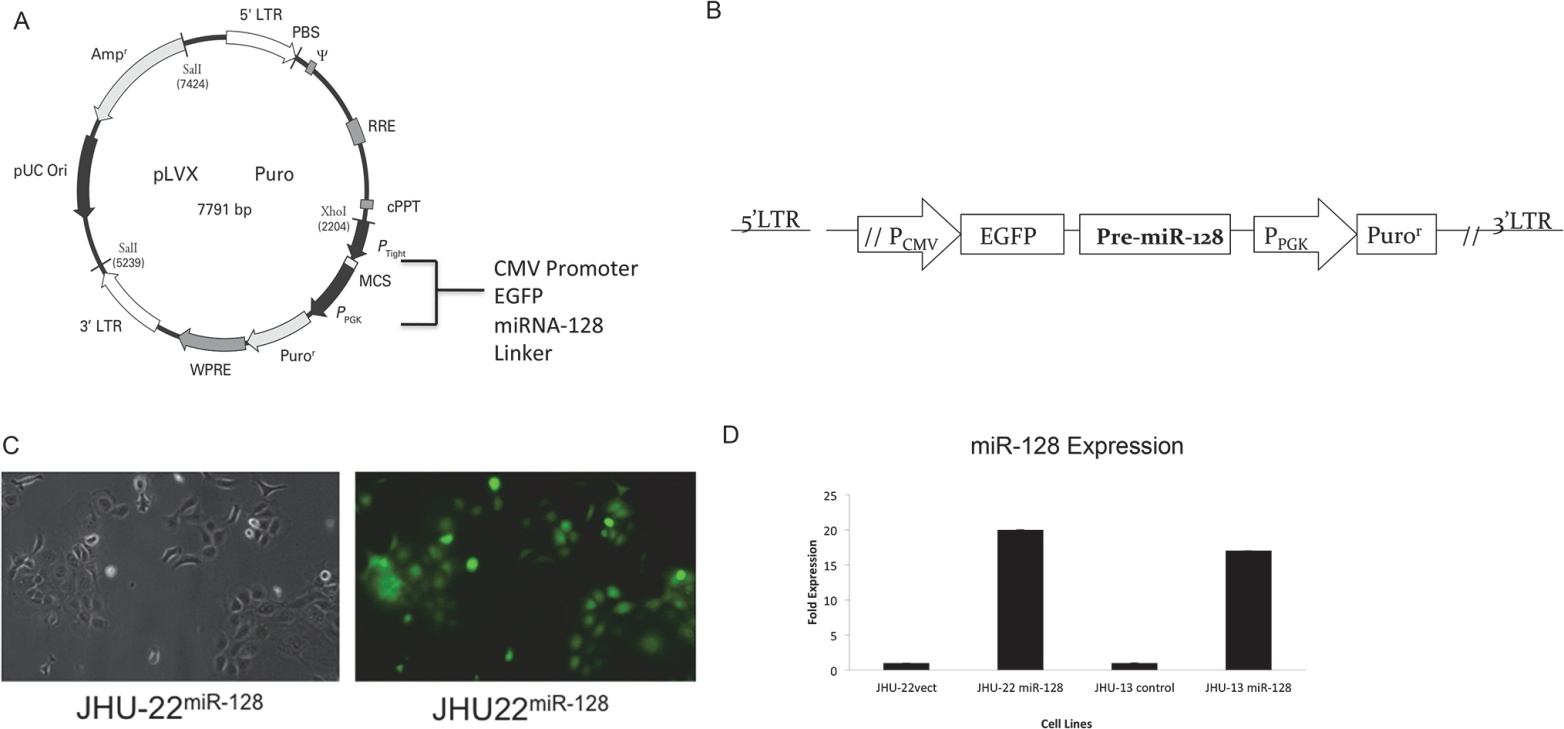
Stable expression of the miR-128 in JHU-22 head and neck squamous cancer cell lines. (A) Schematic representation of the design of the lentiviral miRNA expression vector. (B) Schematic representations of the expression cassette- the P_CMV_ promoter, EGFP, and miRNA precursor-128-1, and a selective cassette of the P_PGK_ promoter and Puro^r^. (C) JHU-22^mi-128^ cells viewed under optic-microscope (left) and fluorescent-microscope (right). (D) Levels of miR-128 in JHU-22^miR-128^ and JHU-13^miR-128^ compared to control vectors in cultured cell lines were determined using QRT-PCR.

This study was proposed to investigate the functions of miR-128 using miR-128 stably transfected HNSCC cell lines. Human HNSCC JHU-13 and JHU-22 cell lines were chosen for the studies because both cell lines have relative low levels of miR-128. The stable transfection was performed using a lentiviral delivery system, which contained an expression cassette of the CMV promoter, EGFP, and miR-128 precursor (Fig. 1A and B). Fig. 1 shows an example for generation of JHU-22^miR-128^ cell line. Hsa-miR-128–pseudotyped lentiviral particles contained a double-stranded 21-nucleotide of ″miR-128 mimic″. The particles were generated by using the Lenti-X HT Packaging System in 293T cells. JHU-22^vect^ control cell line was transfected with the lentiviral particles containing EGFP vector. Enforced miR-128 expression was monitored under fluorescence microscope on the basis of EGFP expression (Fig. 1C) and quantified by QRT-PCR (Fig. 1D). The miR-128 expression levels were increased approximately 16 folds in JHU-13^miR-128^ cells and 19 folds in JHU-22^miR-128^ cells compared to their vector control cells (Fig. 1D).

### Up-regulated miR-128 suppresses the expression levels of its putative target proteins

**Fig 2:**
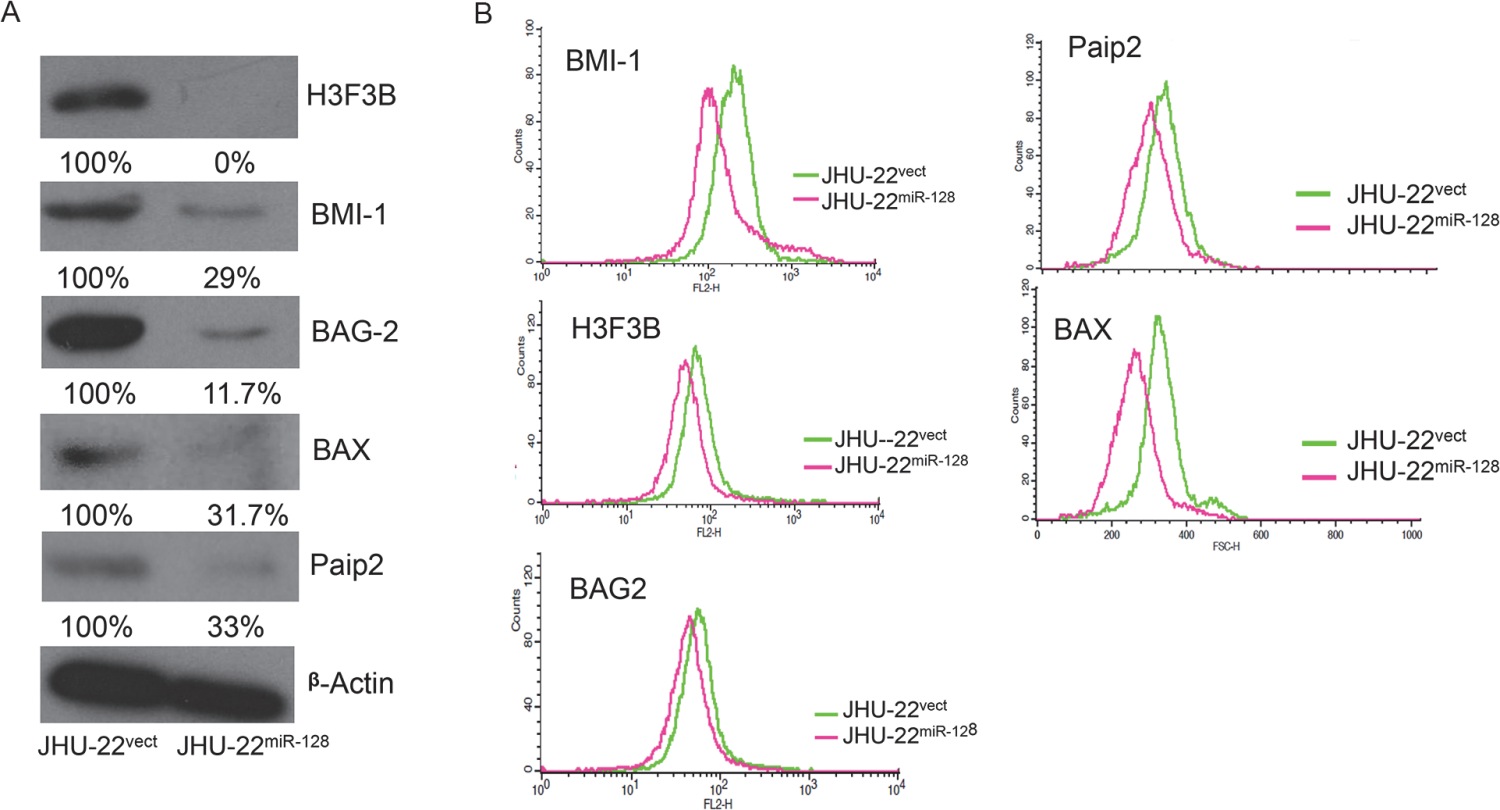
Comparison of expression levels of the putative targets between JHU-22^miR-128^ and JHU-22^vect^. (A) The protein levels of putative targets determined by Western blotting analysis. The amount of protein was normalized relative to the intensity of ß-actin band and was semi-quantified based on relative intensity. (B) The protein levels of putative targets determined by flow cytometry.

The putative targets of miR-128 were first identified using TargetScan 5.0 and Pic Tar microRNAs prediction software that predicted targets with strong and highly conserved miR-128 target sites in the 3′-UTR of targeted mRNA (Table 1). A panel of conserved five targets were selected including the proliferation regulator, BMI-1; pro-apoptotic regulator, BAX; transcription-H3 histones family (H3F3B), BCL-2 associated anthanogene-chaperone (BAG 2), and poly A binding protein interacting protein 2 (PAIP2). Each putative target has a near-perfect binding site in its 3′-UTR for miR-128. The TargetScan’s prediction rates were greater than 94% (Table 1). As expected, the protein levels of these targeted genes were significantly decreased in JHU-22^miR-128^ cells compared with JHU-22^vect^ (Fig. 2). With Western blotting, the protein level of H3F3B was barely detected, BAG-2 protein was reduced more than 80%, and BMI-1, BAX and Paip2 were reduced approximately 70% (Fig. 2A). These findings were further validated with flow cytometry. The flow cytometry analysis revealed a left shift in fluorescence intensity of the targeted proteins in miR-128 transfected cells (JHU-22^miR-128^) compared to the vector control cells (JHU-22^vect^), indicating a decrease in labeled protein levels (BMI-1, H3f3B, BAG-2, BAX, and PAIP2). This was shown to a similar tendency with Western blotting (Fig. 2B and C).

**Table 1:** Summary of sequence alignment of putative and binding capacities targets of miR-128.

Target	TargetScan Human 5.0	Luciferase (Fold Expression) Decrease*	Position	Sequence
**PAIP2**	98%	6.5	42–48 of PAIP2 3’UTR	5' …GUAGCACAAUUUCCA**CACUGUG**A…
**BAG2**	98%	2.0	613–619 of BAG2 3’UTR	5' …CCACCUAUAAUUUAC**CACUGUG**A…
**H3F3B**	97%	9.0	388–394 of H3f3B 3’UTR	5' …AGUCU**GACC**AUACAU**CACUGUG**A…
**BMI-1**	97%	29.0	481–487 of BMI-1 3’UTR	5' …UCUAUGUAGCCAUGU**CACUGUG**A…
**BAX**	94%	5.0	50–56 of BAX 3’UTR	5'…CCACCUAUAAUUUAC**CACUGUG**A…

3′-UTR of PAIP2 (NM_001033112 3′-UTR 42–48), BAG-2 (NM_004282 3′-UTR 631–619), H3f3b (NM_005324 3′-UTR 388–394), BMI-1(NM_005180 3′-UTR 481–487), and BAX (NM_004324 3′-UTR 50–56) were found to be the potential target of miR-128, and carries a complementary site for the seed region of miR-128.

*TargetScan Human 5.2 total context score http://www.targetscan.org

### The binding capacities of miR-128 with its putative targets

**Fig 3:**
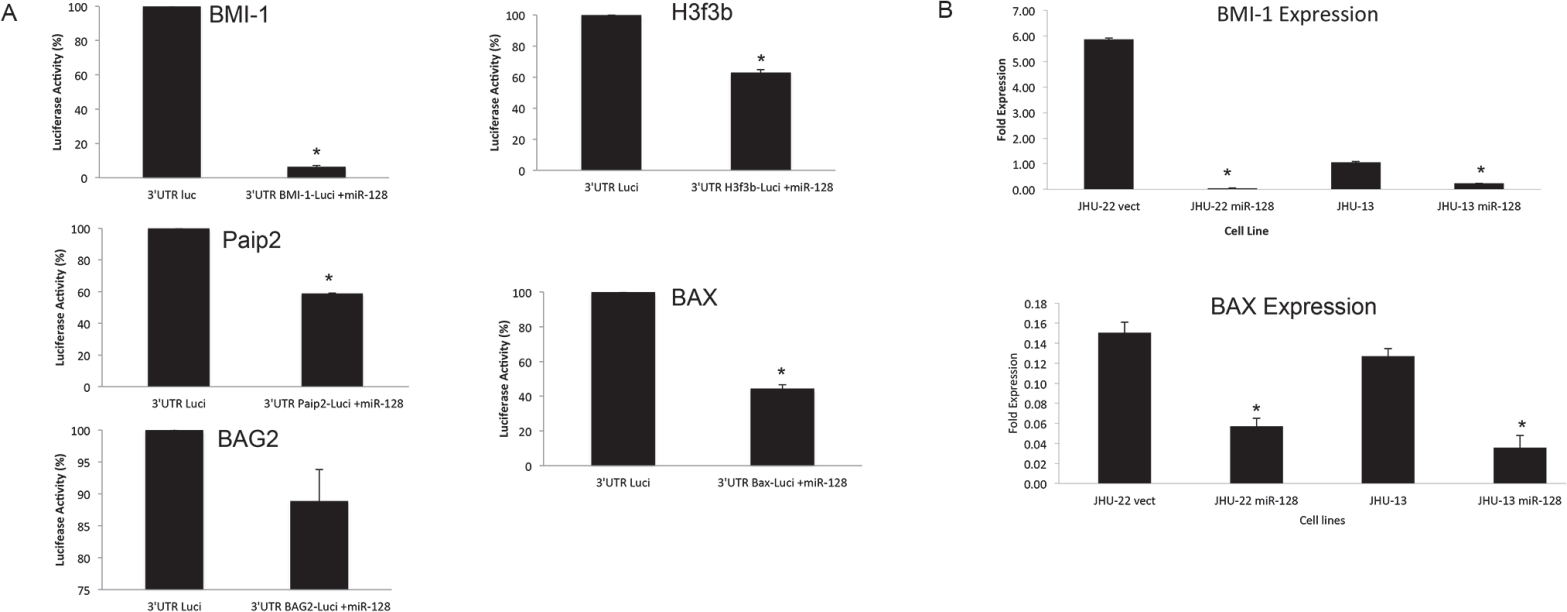
Binding capacity of miR-128 with individual putative targets determined by luciferase report assay. (A) A set of tests included 3’UTR-target-luci vector alone, EGFP-miR-128 vector alone, and combination of 3’UTR-target-luci vector and EGFP-miR-128 vector. The luciferase activity was detected by Xenogen IVIS instrument. The EGFP background in the assay was subtracted from the luciferase activities. The results represented the mean ± SD for 2 independent experiments with triplicates and *P*<0.05. (B) Levels of BMI-1 and BAX mRNA expression in cultured cell lines were determined using QRT-PCR.

We utilized the luciferase report assay to determine the binding capacity of miR-128 with its putative targets. Individual 3′-UTR fragment of targeted mRNA was cloned into the upstream of the luciferase reporter in the vector (3′-UTR-LuciVector), and the binding assay was performed in 293T cells. The luciferase activities were measured after 48 hours’ transfection with individual 3′-UTR-LuciVector or EGFP-miR-128 vector alone, or co-transfected with both vectors in 293T cells. We found that the luciferase activities were significantly reduced in these co-transfected cells compared with transfected 3′-UTR-LuciVector alone except in the co-transfected with 3′-UTR-BAG2-LuciVector (Fig. 3A). The luciferase activities were reduced 29.0 folds in co-transfection with 3′UTR-BMI-1-Luci vector, 9.0 folds in co-transfection with 3′UTR-H3F3B-Luci vector, 6.5 folds in co-transfection with 3′UTR-PAIP2-Luci vector, and 5.0 folds in co-transfection with 3′UTR-BAX-Luci vector (Fig. 3A). The binding capacity of miR-128 towards its putative targets was summarized in Table 1. We further utilized the QRT-PCR to determine the effect of miR-128 in the mRNA levels of BMI-1 and BAX in JHU-13^miR-128^ and JHU-22^miR-128^ cell lines compared to their vector controls (Fig. 3B). BMI-1 mRNA levels were reduced 5 folds in JHU-22^miR-128^ cells and 1 fold in JHU-13^miR-128^ cells compared to their control vector cells. BAX mRNA levels were reduced 0.14 fold in JHU-22^miR-128^ cells and 0.09 fold in JHU-13^miR-128^ cells compared to their control vector cells.

### Up-regulated miR-128 expression inhibits the growth of cultured JHU-13^miR-128^ cells and JHU-22^miR-128^ cells, and JHU-22^miR-128^ tumor xenografts

**Fig 4:**
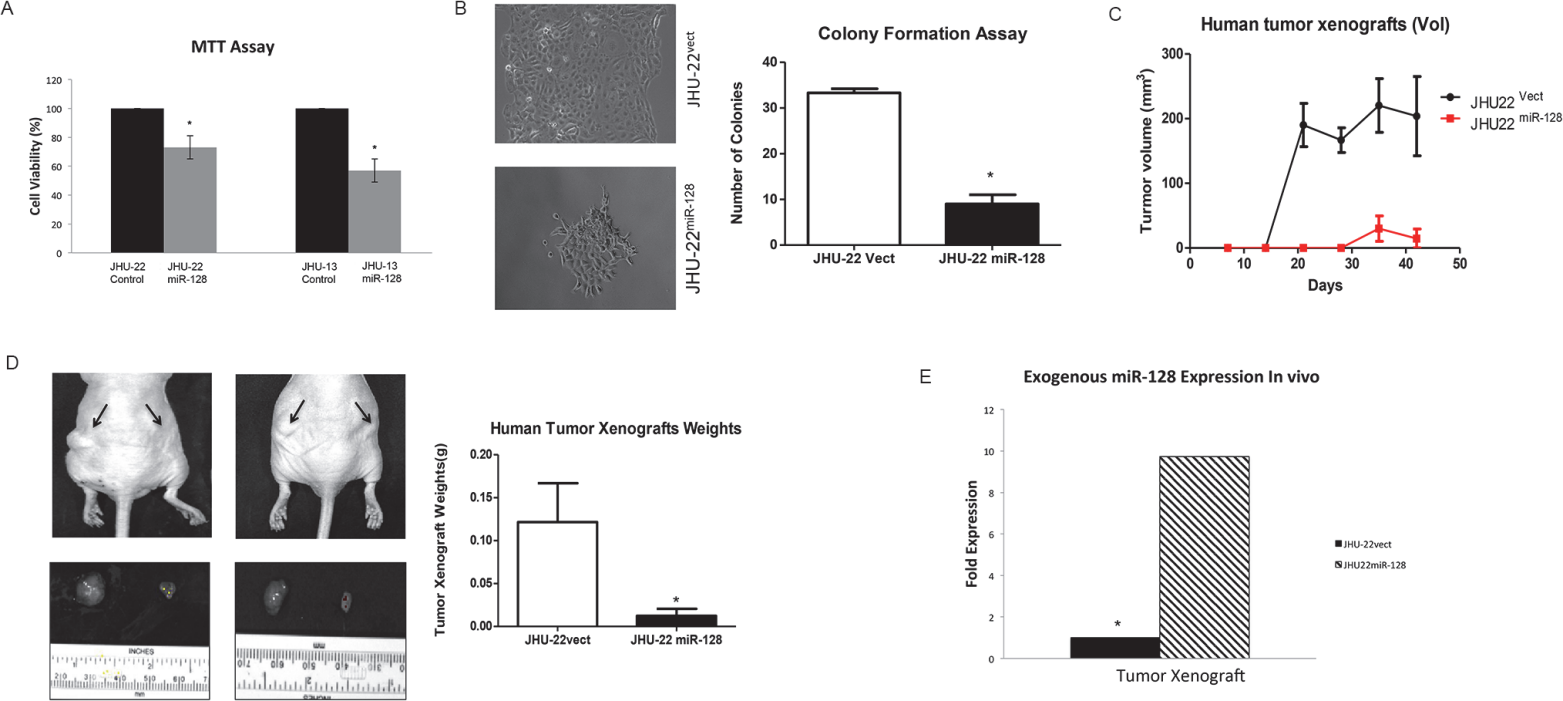
Evaluation of miR-128 effects on cell viability, proliferation and xenograft growth. (A) The cell viability levels were analyzed by MTT. The results represent the mean ± SD of five independent experiments (*P < 0.05). (B) The cell proliferation levels were determined by a colony formation assay. The colonies were viewed under optic-microscope at day-9 (left) and the colony number was counted (right). (C) The day of cell inoculation was the experimental start day and all mice were sacrificed on day 42. (D) Whole body imaging with tumor xenografts JHU-22^vect^ or JHU-22^miR128^ cells, 1 x 10^6^ cells in 100 μl, were inoculated subcutaneously into the lower back of the mice. JHU-22^vect^ cells on the left and JHU-22^miR-128^ cells on the right. The arrows indicated the tumor locations. Tumor mass was measured on the final experimental day immediately after the tumor tissue was removed from the mouse by surgical excision. Results are presented as the mean ± SD, n = 10. (E) Levels of miR-128 in cultured cells and tumor xenografts were determined using QRT-PCR.

The effects of miR-128 on the regulation of cell viability and colonigenic potential were evaluated using MTT (Fig. 4A) and colonigenic formation assays (Fig. 4B), respectively, in cultured JHU-13^miR-128^ and JHU-22^miR-128^ cells. The results from MTT assay showed that unregulated miR-128 expression in JHU-13^miR-128^ and JHU-22^miR-128^ cells led to approximately 22% to 43% reduction of cell viability (Fig. 4A). In addition, JHU-22^miR-128^ cells displayed significant decreased capability to form colonies, showing a low number of colonies in comparison to JHU-22^vect^ cells (Fig. 4B). The colony forming ability of JHU-22^miR-128^ was 55% less than that of JHU-22^vect^ cells. In addition, phenotypic changes were observed in JHU-22^miR-128^ cells, which appeared to have round bodies resembling characteristics of astrocyte-star shaped form and were smaller than the control JHU-22^vect^. We analyzed cell cycle regulation, focusing on the changes in S-phase compared to G1 and G2 after 20 hours and 24 hours; however we did not observe significant changes between the control vector and miR-128 in JHU-22 cell lines (S1 and S2 Fig.). We further investigated the anticancer function of miR-128 in tumor xenograft animal model (Fig. 4C and D). In order to limit individual mouse effects, both JHU-22^vect^ and JHU-22^miR-128^ cells were subcutaneously inoculated into a Balb/c athymic nude mouse. A growth curve of tumor xenograft was showed in Fig. 4C that JHU-22^vect^ tumor xenografts were able to visualize two weeks after inoculation and JHU-22^miR-128^ xenografts appeared four weeks after inoculation (Fig. 4C). The average tumor weight in JHU-22^vect^ group was significantly higher than JHU-22^miR-128^ group (0.15 g *vs*. 0.01 g, *P* < 0.05) (Fig. 4D). The miR-128 level in JHU-22^miR-128^ tumor xenografts was maintained approximately 10-fold higher than in its control group (Fig. 4E).

### Up-regulated miRNA-128 expression regulates the levels of cell growth and apoptotic mediators

**Fig 5:**
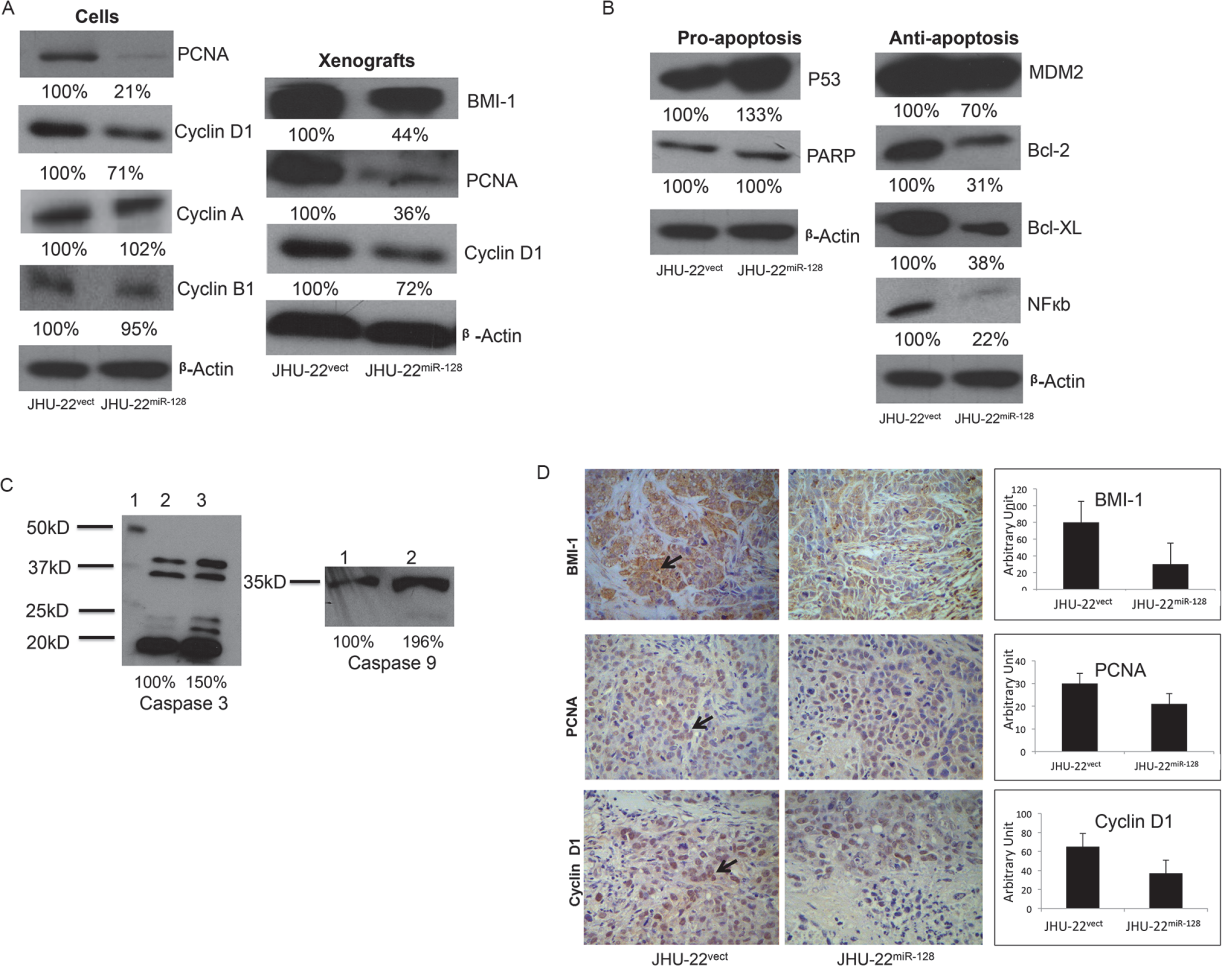
Effects of miR-128 expression on the profiles of cell growth and apoptotic indicators. (A, B, C) JHU-22^vect^ and JHU-22^miR-128^ protein levels in cell lines were evaluated by Western blotting analysis. The amount of protein was normalized relative to the intensity of ß-actin band and was semi-quantified based on relative intensity. (D Immunohistochemistry (IHC) effects of miR-128 expression in xenograft tissues. The levels of BMI-1, PCNA and cyclin D1 in the xenograft tissues were analyzed using IHC analysis. All IHC measurements were based on semi-quantitative scoring.

The protein profiles of cell growth and apoptotic mediators were analyzed by Western blotting and immunohistochemistry. Overall, the cell cycle and proliferation indicators including PCNA, cyclin D1 and BMI-1, were significantly down-regulated by approximately 30 to 80% in JHU-22^miR-128^ cells and JHU-22^miR-128^ xenograft tissue compared to the control groups (Fig. 5A). The pro-apoptotic regulators including p53 and caspase-9 were significantly up-regulated approximately 20 to 30%. There were no notable changes with PARP; however, the anti-apoptotic proteins including MDM2, Bcl-2, Bcl-XL, and NFkb were dramatically down-regulated approximately 30 to 80% in cultured JHU-22^miR-128^ cells compared with JHU-22^vect^ cells (Fig. 5B). Caspase 3 is a cytosolic protein found in cells as an inactive proenzyme at 32 kDa. It is activated by proteolytic cleavage into a 20kDa active subunit only when cells are undergoing apoptosis (Fig. 5C). Inactive Caspase 9 at 47kDa is also activated by cleavage into a 35kDa active subunit (Fig. 5C). Fig. 5D shows a comparison of the expression profiles of protein index between JHU-22^miR-128^ and JHU-22^vect^ tumor xenografts. Again, the levels of BMI-1 (30/80), PNCA (21/30) and cyclin D1 (37/65) were significantly down-regulated in the JHU-22^miR-128^ group compared with the control JHU-22^vect^ group.

## Discussion

miRNAs are recognized as a class of gene modulators regulating various physiological and pathological events. The miRNAs are predicted to regulate the expression of over 30% of all genes and may account for some of the aberrant gene expression in cancer cells. miR-128 is uniquely encoded by two distinct genes, miR-128a and miR-128b, which are processed into an identical mature sequence. miR-128a and miR-128b are both intronic, embedded in the R3HDM1 gene on chromosome 2q21.3 and ARPP21 gene on chromosome 3p22, respectively. The molecular and cellular functions of miR-128 are expressed in numerous pathways and organs within the body [23]. TargetScan 5.0 has categorized and predicted targets of miR-128 in both conserved and non-conserved sites. Intriguingly, miR-128 is shown to be down-regulated with age, affecting genetic diseases, and is shown to function as a tumor suppressor. Specifically, miR-128 is shown to block major singling pathways such as ERK and AKT in tumor development, resulting in the inhibition of proliferation, metastasis and angiogenesis in non-small cell lung cancer [24]. Conversely, endometrial cancers expression miR-128 is shown to be up-regulated [25]. Moreover, overexpression of miR-128 has been associated with reduced cell growth in glioma tissue and cell lines [19,26,27]. The observations from this study, that up-regulation of miR-128 inhibited HNSCC growth through directly mediating its targets Paip2, BAG-2, H3F3B, BMI-1, and BAX in proliferation and apoptotic pathways, support that miR-128 functions as a tumor suppressor.

All of the targeted mRNAs have a complementarity 3′-UTR region, which can pair with miR-128 to impede the translation of targeted mRNA resulting in a down-regulated protein level. BMI-1, H3F3B and Paip2 proteins are involved in cell proliferation. BMI-1 is a polycomb ring finger oncogene regulating the p16 and p19, cell cycle inhibitor genes. BMI-1 is also necessary for efficient self-renewing cell divisions of adult hematopoietic stem cells as well as adult peripheral and central nervous system neural stem cells [28]. Recent reports indicate that BMI-1 can be rapidly recruited to sites of DNA damage [29]. H3F3B constitutes the predominant form of histone H3 in non-dividing cells and is incorporated independently into chromatin of DNA synthesis. H3F3B plays a central role in transcription regulation, DNA repair/replication, and chromosomal stability [30,31]. In addition, BMI-1 represses transcription through chromatin modification [32]. Interestingly, both BMI-1 and H3F3B showed a higher binding rate with miR-128 among these five potential targets analyzed with luciferase report assay (Table 1). Simultaneously, the protein levels of BMI-1 and H3F3B were largely reduced in JHU-22^miR-128^ cells. We found that reduction of BMI-1 and H3F3B expression led to diminish JHU-22^miR-128^ cell proliferation and xenograft growth (Fig. 4), Furthermore, we observed significant reduction in cell growth of JHU-13^miR-128^ (Fig. 4A). We confirmed miR-128 binding capacity to the 3’UTR of the mRNA of BMI-1 (Fig. 3A). We found significant reduction of BMI-1 expression in JHU-22^miR-128^ and JHU-13^miR-128^ compared to the control vectors. Additionally, we also found that the expression levels of cell proliferation-related regulators were altered, showing reduced protein levels of PCNA and cyclin D1 in JHU-22^miR-128^ cells. PCNA acts as a progressive factor for DNA polymerase and a cell cycle index that essentially binds to cyclin D-CDK4, cyclin E-CDK2 and cyclin A-CDK2 complexes for the progression from G1 to S phase in the cell cycle. Cyclin D1 is a another key regulator for the G1 to S transition and tumor progression that activates cyclin D-CDK4 complex to enhance the transcription of cell cycle related genes [33]. Paip2 might be another direct target of miR-128, since the protein level of Paip2 was reduced more than 60% in the JHU-22^miR-128^ cells (Fig. 2A). Paip2 acts as a repressor in the regulation of translation initiation of poly (A)-containing mRNAs. Paip2 interacts with the poly (A)-binding protein (PABP), which prevents poly A binding to the poly A tail of mRNAs and thereby inhibits cap-dependent and cap-independent translocations through the circularization of mRNA [34]. Controlling translation of mRNA is crucial for the homeostasis of a cell. Its dysregulation can facilitate the development and progression of many diseases including cancer. Consistent with these reports, our data supports that dysregulation of miR-128 is involved in the HNSCC growth and progression. Enforced miR-128 expression is of potential value in HNSCC therapy.

Our data also suggested that miR-128 is able to balance the ratio of protein expression between the anti-apoptotic regulator BAG-2 and the pro-apoptotic regulator BAX. The protein level of BAG-2 was extremely high in the HNSCC JHU-22 cells (Fig. 2A), however, up-expressed miR-128 in the JHU-22^miR-128^ cells was able to attenuate BAG-2 protein significantly, even though BAG-2 had a relative lower binding capacity with miR-128 compared to BMI-1, H3F3B, or BAX (Table 1). The protein ratio of BAG-2 *vs*. BAX was found to be 2:1 in the JHU-22^vect^ cells; however, the ratio was significantly changed in the JHU-22^miR-128^ cells that BAG-2 *vs*. BAX was 0.7:1. Its effects are seen at a higher capacity compared to BAX. Overall, our results imply that miR-128 leads HNSCC cell apoptosis (Figs. 2 and 5, and Table 1). BAG-2 is a chaperone regulator, which binds to the heat shock protein (Hsp70/Hsc) that regulates this chaperone activity and apoptosis. Malfunction in this regulator has been shown to result in protein mis-folding and cells to survive under lethal condition [35]. On other hand, BAX gene is the first identified pro-apoptotic member of the Bcl-2 protein family [36]. BAX induces apoptosis *via* regulating the permeabilization of mitochondrial outer membrane [37]. The protein level of BAX was low in the control JHU-22^vect^ cells. Moreover, a recent report has shown that miR-128 exerts pro-apoptotic effects *via* directly inhibiting SIRT1 expression to lead an increase in acetylated p53 and its transcriptional targets [38]. As expected, we found the levels of pro-apoptotic regulators, such as p53, and caspases 3 and 9 (Fig. 5B and C) were elevated, and the levels of anti-apoptotic regulators, such as Bcl-XL, Bcl-2, NFκb and MDM2 significantly decreased in JHU-22^miR-128^ cells (Fig. 5B). Pro-survival proteins, Bcl-2 and Bcl-XL, prevent the release of cytochrome C from the mitochondria. This leads to caspase activation (e.g. caspase 3, caspase 9, etc). Another possibility is that miR-128 expression induces apoptosis by disrupting the mitochondrial membrane potential which would cause the release of cytochrome C and increase caspase activity.

Several conclusions could be drawn based on the data reported herein. First, miR-128 had the capacity to directly bind to the 3’-UTR region of these targeted mRNAs of H3f3b, BMI-1, PAIP2, BAG-2, and BAX. Secondly, miR-128 was able to suppress mRNA and protein levels of H3f3b, BMI-1, PAIP2, BAG-2, and BAX expression, but the degree of inhibition varied. Third, up-regulated miR-128 expression inhibited HNSCC cell growth and promoted HNSCC cell apoptosis partially through mediating its direct targets. Lastly, our comprehensive analysis elucidated a possible mechanism of miR-128 function as a tumor suppressor, and suggested great clinical value of miR-128 as a therapeutic target and indicator. The present data indicates that miR-128 is involved in multiple signal pathways that are associated with HNSCC progression and growth. Further studies are warranted to confirm the expression and function of miR-128 with human tumor samples of HNSCC and other pathological types.

## Supporting Information

S1 Fig Cell Cycle Analysis JHU-22^vect^ and JHU-22^miR-128^ Cell Lines 20hrs. Cell cycle was analyzed by flow cytometry after cells were treated with FBS 20 hours. The distributions of cells in G1, S, and G2 phases are shown for JHU22^vect^ (A) and (B) miRNA transfected JHU-22^miR-128^ cell lines. The results represent the mean ± SD from independent experiments performed in triplicate.
(PDF)

S2 Fig Cell Cycle Analysis JHU-22^vect^ and JHU-22 ^miR-128^ Cell Lines 24hrs. Cell cycle was analyzed by flow cytometry after cells were treated with FBS 24 hours. The distributions of cells in G1, S, and G2 phases are shown for JHU22^vect^ (A) and (B) miRNA transfected JHU-22^miR-128^ cell lines. The results represent the mean ± SD from independent experiments performed in triplicate.
(PDF)
