# Supplementary material for: Functions of MiRNA-128 on the Regulation of Head and Neck Squamous Cell Carcinoma Growth and Apoptosis
Source: PLoS One. 2015 Mar 12;10(3):e0116321. doi: 10.1371/journal.pone.0116321 (PMC4357443; doi:10.1371/journal.pone.0116321)
Supplement: S2 Fig — Cell cycle was analyzed by flow cytometry after cells were treated with FBS 24 hours. The distributions of cells in G1, S, and G2 phases are shown for JHU22vect (A) and (B) miRNA transfected JHU-22miR-128 cell lines. The results represent the mean ± SD from independent experiments performed in triplicate. (PDF) [file pone.0116321.s002.pdf]

Supporting Information

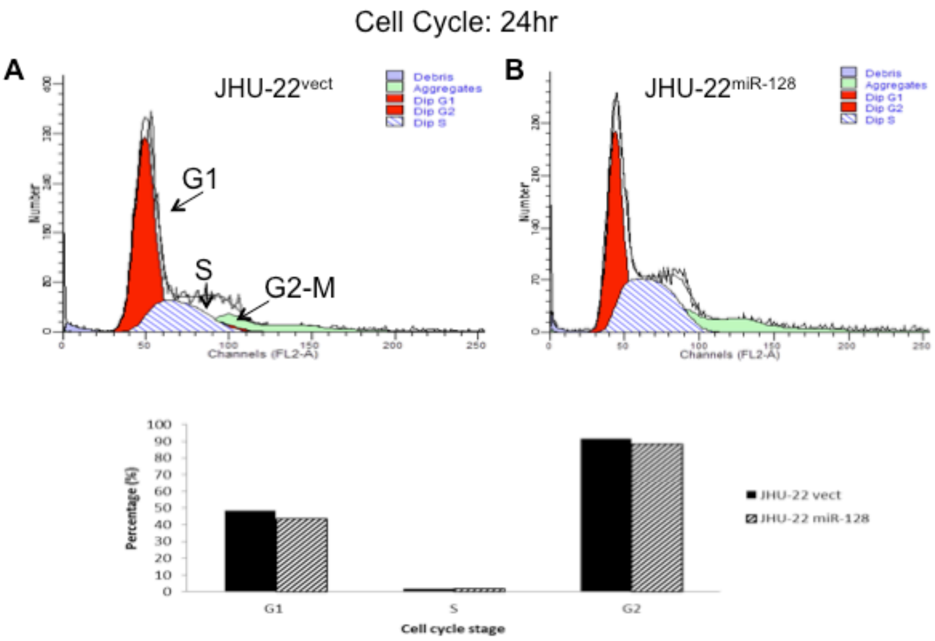

Figure S2.

Cell Cycle Analysis JHU-22^{vect} and JHU-22$^{miR-128}$ Cell Lines 24hrs.

Cell cycle was analyzed by flow cytometry after cells were treated with FBS 24 hours. The distributions of cells in G1, S, and G2 phases are shown for JHU-22^{vect} (A) and (B) miRNA transfected JHU-22$^{miR-128}$ cell lines. The results represent the mean ± SD from independent experiments performed in triplicate
